# Bone Marrow Negative Visceral Leishmaniasis in an Adolescent Male

**Published:** 2013

**Authors:** S Jetley, S Rana, S Khan, JS Zeeba, MJ Hassan, P Kapoor

**Affiliations:** 1 Department of Pathology, Hamdard Institute of Medical Sciences and Research, New Delhi, 110062, India; 2 Department of Medicine, Hamdard Institute of Medical Sciences and Research, New Delhi, 110062, India

**Keywords:** Visceral, Leishmaniasis, Splenic, Aspirate, India

## Abstract

Visceral Leishmaniasis or Kala Azar is endemic in certain regions of India. In endemic areas, the constellation of fever, progressive weight loss, weakness, pronounced splenomegaly, anemia, leukopenia, and hypergammaglobulinemia is highly suggestive of visceral leishmaniasis. Demonstration of the parasite in liver, splenic or bone marrow aspirates is confirmatory. We present a case in which *Leishmania donovani* (LD) bodies were demonstrated on splenic aspirate. We were unable to demonstrate LD bodies on bone marrow aspiration.

## Introduction

*Leishmania* infections are worldwide in distribution. Visceral leishmaniasis (VL) or kala-azar is widespread in tropical and subtropical regions of Africa, Asia, the Mediterranean, southern Europe and south and Central America. Ninety percent of cases are reported from Bangladesh, India, Nepal and Sudan (1). Around 1,00,000 new cases occur each year and at least 200 million people are at risk of infection (2). In India, it is a major public health problem in the states of Bihar, Jharkhand, West Bengal, and parts of Eastern Uttar Pradesh (3). *Leishmania*-HIV coinfection is an emerging disease especially in southern Europe, where 25 to 70% of adults with VL also have AIDS (4).

Visceral leishmaniasis is characterized by prolonged fever, massive splenomegaly, weight loss, progressive anemia, pancytopenia, and hypergammaglobulinemia, and can be complicated by serious infections. It is the most severe form of leishmaniasis and, if left untreated, is usually fatal. In the Indian subcontinent, the disease is almost exclusively caused by *L. donovani* and transmitted by phlebotomine sandflies. A confirmatory diagnosis is achieved by demonstrating *Leishmania* parasite in tissues (5). Splenic aspirate, has a high sensitivity, although it carries a risk of fatal haemorrhage in inexperienced hands.

Our patient had classical presentation of VL which was confirmed by demonstration of the amastigote forms on splenic aspiration.

## Case Report

Our patient, a fifteen year old male, resident of Bihar had complaints of longstanding fever, abdominal distention, weight loss and generalized weakness since ten months. He then reported to the local district hospital where the rK39, rapid diagnostic test for kala azar was done and found positive. He was given three injections of amphotericin deoxycholate to which he reacted with fever and hypotension. No further medication was given or investigations carried out. The parents were migrant laborers and the boy was brought to the outpatient department of our hospital in New Delhi after another two months when symptoms persisted.

**Fig. 1:**
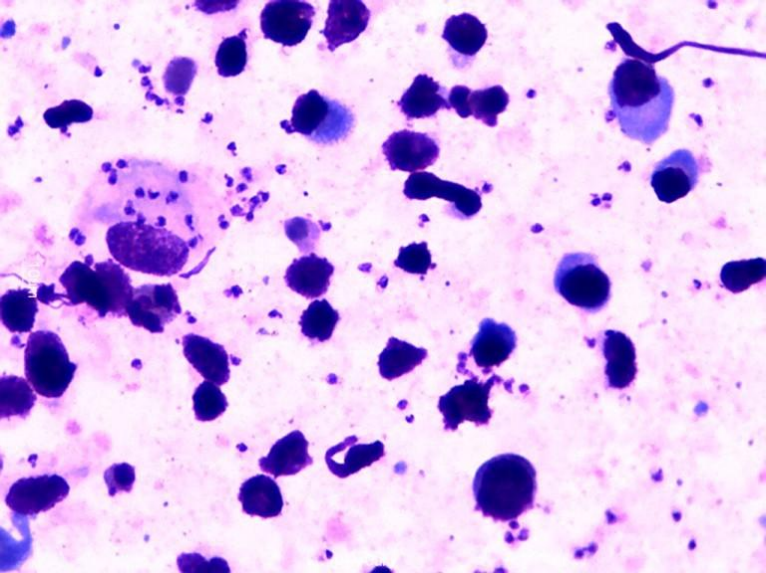
Microphotograph showing both intracellular and extracellular LD bodies in splenic aspirate (Giemsa, 100X)

On examination, he was febrile, undernourished, with massive splenomegaly and hepatomegaly. Hematological investigations showed moderate pancytopenia, Hb 8.3g/dl, TLC 2000/mm^3^, platelet count 75,000/mm^3^. rK 39 Rapid Diagnostic test was repeated and was negative. ELISA tests for HIV, HbsAg and HCV were all negative. Mantoux test was also negative. Liver and renal function tests were within normal limits. Bone marrow aspiration showed reactive plasmacytosis (10%) and no *Leishmania donovani* (LD) bodies were seen. Napier's aldehyde test was performed which was strongly positive. In view of the strong clinical suspicion of VL and the positive aldehyde test, splenic aspiration was done. Aspirates showed both intracellular as well as extracellular amastigote forms of LD bodies, thus confirming VL (Fig. 1). A combination therapy was started with pulse liposomal amphotericin B (10 mg/kg body weight) i.e. 250 mg as a single dose therapy followed by tablet miltefosine 50mg O.D. for one week. Patient responded well to the therapy. He became afebrile with improvement of the blood counts and regression of the spleen size.

## Discussion

Visceral leishmaniasis caused by either *Leishmania donovani* (East Africa and India) or *Leishmania infantum* and *Leishmania chagasi* (Europe, North Africa and South America). It is a major opportunistic infection in patients with AIDS and epidemiological data has shown that kala-azar has re-emerged from a near eradicated status.(4) Bite of the vector sandfly transmits the disease to humans when the parasite is engulfed by macrophages and eventually infects the entire reticuloendothelial system resulting in the classical picture of VL.

Diagnosis of VL can often be problematic as clinical features are shared by other commonly occurring diseases such as malaria, typhoid and tuberculosis. Direct parasitological evidence is the bed-rock on which the diagnosis of kala-azar rests. LD bodies can be demonstrated in spleen, bone marrow, lymph node or buffy coat smears. Bone marrow and splenic aspirates are used routinely. Bone marrow aspiration is a safer, but less sensitive method in the diagnosis of VL as compared to splenic aspiration. Splenic aspirate, though associated with risk of fatal hemorrhage in inexperienced hands, is one of the most valuable methods for diagnosis of kala-azar, with a sensitivity exceeding 95% (6). Some experienced physicians even prefer splenic aspiration over a bone marrow aspirate as it is an easy to perform, short procedure without serious complications (7, 8). It is less painful than bone marrow aspiration which has a lower sensitivity of approximately 60-85% (6). The only risk of splenic puncture is bleeding from a soft and enlarged spleen. Sunder et al reported fatal bleeding only twice in 9,612 splenic aspirate procedures performed over a period of 10 years (6). Splenic aspiration with its high sensitivity rates often acquires a pivotal role in initiating specific anti-leishmanial therapy in relapsed cases, when rK 39 strip test and the bone marrow examination are negative for VL, as was seen in the present case.

As in other diseases, specific serodiagnostic tests for antibody detection in the diagnosis of kala-azar have also been employed. Antileishmanial antibodies are typically present in high titre in immunocompetent patients with visceral leishmaniasis. Enzyme-linked immunosorbent assay (ELISA) tests with a kinesin-like antigen, have good sensitivity and specificity for the diagnosis of visceral leishmaniasis in immunocompetent persons and titers correlate directly with disease activity (9). However its practical application in field conditions has not taken off as sophisticated laboratory setup and skilled man-power is required. DAT (direct agglutination test is a very suitable and reliable serological test due to its high sensitivity and specificity. DAT helps in bringing down the mortality caused due to delay in the diagnosis and treatment (10, 11).

rK 39 rapid diagnostic test is a ready to use strip test which has found wide acceptability in the Indian subcontinent (12). As per the recommendations of the WHO Expert committee on kala-azar, it was concluded that this test is comparable to parasitology in terms of sensitivity and can replace parasitology as the basis for a decision to treat visceral leishmaniasis at peripheral health centers in endemic areas (13). This probably was the basis on which specific treatment was initiated in this case at the district hospital, though the patient reacted adversely and treatment was incomplete leading to a relapse. Persistence of antibodies well beyond cure and inability of some patients to produce enough antibodies are inherent limitations with all antibody-based diagnostics, including the rK 39 strip test. These factors limit the utility of this test in predicting cure or diagnosing relapses. In the present case, rK 39 Rapid diagnostic test when repeated at our hospital was negative as also was the bone marrow examination for LD bodies. In spite of these limitations, the rK39 immunochromatographic strip test is indeed a boon in confirming VL in endemic areas. It can be performed by paramedics in field conditions and turns positive early in the course of the disease besides being the only available diagnostic test for VL with acceptable sensitivity and specificity levels which is also simple and inexpensive (1 to 1.5 US dollars for each strip). Contrastingly, in Europe, where HIV co-infected with VL is common, a lower sensitivity for the rK 39 Rapid diagnostic test has been reported (14).

In a prospective study of fifty adult patients of kala-azar in Mymensingh, Bangladesh, splenic and bone marrow aspiration was done simultaneously in all cases to compare the sensitivity of both the procedures (7). The authors concluded that splenic aspiration was a more sensitive procedure than bone marrow aspiration besides being more acceptable to patients as it was less painful. They reported LD bodies in 90% of splenic aspirates as compared to 72% positivity of bone marrow aspirates. Baro et al. in their study of fresh as well as relapsed cases of VL noted that as compared to bone marrow aspiration, splenic aspirates continued to show LD bodies for a longer time during the treatment follow-up but once negative, no relapses occurred during a 6 month observation period (8).

## Conclusion

Parasitological diagnosis with demonstration of the amastigote forms in tissues remains the “gold standard” among all laboratory tests. In experienced hands, splenic aspiration is indeed the procedure of choice, when all clinical parameters point towards VL and the more routinely employed tests like strip test for kala-azar and bone marrow aspiration are negative, as was seen in our case.
